# Chaperonin (HSP60) and annexin-2 are candidate biomarkers for non-small cell lung carcinoma: Erratum

**DOI:** 10.1097/MD.0000000000007317

**Published:** 2017-06-23

In the article, “Chaperonin (HSP60) and annexin-2 are candidate biomarkers for non-small cell lung carcinoma”,^[1]^ which appeared in Volume 96, Issue 6 of *Medicine*, “adenocarsinoma” is written incorrectly as “adenokarsinoma.” The legends appear correctly below.

Figure 2. Adenocarsinoma macroscopic view.

Figure 4. Adenocarsinoma microscopy view.

Figure 6. Adenocarsinoma spot samples in MALDI spectrometry. MALDI  =  matrix assisted laser desorption ionization.
